# Chromosomal Rearrangements and Chromothripsis: The Alternative End Generation Model

**DOI:** 10.3390/ijms24010794

**Published:** 2023-01-02

**Authors:** Daniel de Groot, Aldo Spanjaard, Marc A. Hogenbirk, Heinz Jacobs

**Affiliations:** 1 Division of Tumor Biology and Immunology, The Netherlands Cancer Institute, Plesmanlaan 121, 1066 CX Amsterdam, The Netherlands; 2 Agendia NV, Radarweg 60, 1043 NT Amsterdam, The Netherlands

**Keywords:** Alternative End Generation (AEG), Alternative End Joining (AEJ), chromosomal rearrangements, chromothripsis, DNA double-strand break repair

## Abstract

Chromothripsis defines a genetic phenomenon where up to hundreds of clustered chromosomal rearrangements can arise in a single catastrophic event. The phenomenon is associated with cancer and congenital diseases. Most current models on the origin of chromothripsis suggest that prior to chromatin reshuffling numerous DNA double-strand breaks (DSBs) have to exist, i.e., chromosomal shattering precedes rearrangements. However, the preference of a DNA end to rearrange in a proximal accessible region led us to propose chromothripsis as the reaction product of successive chromatin rearrangements. We previously coined this process Alternative End Generation (AEG), where a single DSB with a repair-blocking end initiates a domino effect of rearrangements. Accordingly, chromothripsis is the end product of this domino reaction taking place in a single catastrophic event.

## 1. Introduction

Chromothripsis (‘chromo’, from chromosome, and ‘thripsis’, meaning to shatter into pieces) defines a genetic phenomenon where up to hundreds of clustered chromosomal rearrangements arise in a single catastrophic event involving only one or a few chromosomes. Amongst multiple novel insights, NGS uncovered chromothripsis [1], a term initially coined by Stephens et al. [2], along with chromoanasynthesis and chromoplexy as new types of complex genomic rearrangements and instabilities grouped under the term chromoanagenesis, by Holland et al. [3]. Though these types of catastrophic chromosomal phenomena are very similar, they display unique features in the cause or scale of the events. Chromothripsis in general is characterized by a set of distinct features compared to chromoanasynthesis and chromoplexy. Korbel et al. have set out to document these features and set up criteria for chromothripsis, based on the assumption that rearrangements arise through a single catastrophic event, instead of a model where these rearrangements happened progressively [4]. These criteria are defined such that statistical testing of the rearrangements is possible and to test if they occur through chromothripsis [1]. Their molecular criteria signifying chromothripsis are: (1) clustering of the breakpoints, (2) the oscillation of copy number states between one and two copy numbers in the affected region, (3) the loss and retention of heterozygosity is interspersed, (4) only a single haplotype is affected, (5) a randomness of the rearrangement of fragments, and finally (6) the joining of the breakpoints should enable the “walking” of the derivative chromosome [4]. Interestingly, the shattering event in chromoanagenesis is generally thought to occur within a single cell cycle [5], and chromothripsis specifically is thought to happen through massive locally restricted chromosome rearrangements which are acquired in a single catastrophic event [2,3]. These rearrangements can lead to chromosomal amplification and overexpression of oncogenes, but can also lead to the loss of genomic material, potentially causing the deletion of tumor suppressor genes, thus initiating or promoting tumorigenesis [2,6,7,8,9,10]. Indeed, chromothripsis has been linked to tumor initiation and progression in many tumor types, such as multiple myeloma [11,12,13,14,15], pancreatic ductal adenocarcinoma [16,17], gastric cardia adenocarcinoma [18] and breast cancer [19,20,21,22], as well as other cancer types [23,24,25]. Recent pan-cancer analyses using whole-genome sequencing (WGS) analyses revealed its widespread prevalence by having more than thirty percent of the tumors feature evidence of chromothripsis, a much larger number than previously thought [26]. The impact and relevance of chromothripsis is further demonstrated by its role in causing developmental defects and its presence in a range of organisms, from plant to human [27,28,29,30,31].

Though there is a clear consensus based on NGS data that chromothripsis involves hundreds of seemingly random rearrangements, the underlying mechanism(s) of chromothripsis remain a matter of debate. Most models on the origin of chromothripsis imply the existence of tens to hundreds of DNA double-strand breaks (DSBs) preceding chromatin reshuffling [2,10,32,33,34,35]. What these models have in common is that shattering is often accomplished through a set of processes, such as segregation of chromosomes into micronuclei [33,34], abortive cellular processes [35,36] or by massive local exogenous genomic insults [37,38], as discussed in this report. It is unlikely, however, that due to the randomness of endogenous or exogenous stressors, tens to hundreds of DSBs arise simultaneously in a locally confined region of the genome.

Moreover, in cells in which a high number locally confined DSBs would arise, apoptosis would need to be effectively evaded considering the high toxicity of DSBs [39,40,41,42]. Furthermore, while evasion of apoptosis and increased DNA damage may be more likely in cancer cells, this is highly unlikely given the fact that chromothripsis is found in non-transformed cells [27,28,29,30,31]. The extremely low numbers of DSBs that occur in normal cells per day [43] makes it unlikely that hundreds of DSBs arise in a confined region.

We previously observed a preference of DNA ends to rearrange with proximal, accessible, i.e., open chromatin, structures [44]. Here, we propose that this phenomenon can result in chromothripsis, in a model termed Alternative End Generation (AEG). Accordingly, a DNA end of a single DSB rearranges preferentially with an accessible loop in the proximal chromatin, as opposed to the other end of the single DSB due to, e.g., a lesion that blocks extensive end resection or strand invasion, required for homologous recombination (HR). Failure to execute accurate HR favors other, intrinsically mutagenic DSB repair pathways such as nonhomologous end joining (NHEJ) and alternative end joining (Alt-EJ). When the break end fuses with the proximal and accessible chromatin by alternative end joining (Alt-EJ) and related mutagenic DSB repair processes, different repair signatures, including microhomology mediated end-joining at the fusion sites are expected. Accordingly, these signatures are expected to lack long homology tracks characteristic for HR. This notion is consistent with the observation that chromothripsis does not depend on HR but rather processes that do leave behind repair signatures at the break sites characterized by microhomology, as well as small insertions or deletions. Chromatin that fuses with a single DSB DNA end generates a new single DNA end. If this newly generated DNA end cannot be ligated immediately to the blocking end, a domino effect arises, where chromothripsis is the reaction product of consecutive, locally confined AEGs in proximal, accessible chromatin loops (see graphical abstract).

## 2. Chromothripsis: Molecular Characteristics

Like Kataegis, a localized hypermutation process where C > G substitutions within TpCpN trinucleotides accumulate [45], chromothripsis is an exception to the general dogma that mutations are acquired stochastically over relatively long time periods [46,47]. In line with this, Monte Carlo simulations predicted that the rearrangements in chromothripsis involve only a single or a few chromosomes and are likely acquired in a single catastrophic event during cancer development [2]. In contrast, more comprehensive simulations of the same cancer genomes showed that, statistically, chromothripsis could arise conventionally, i.e., as a reaction product of progressive rearrangements [48]. Here, the authors argue that the shattering and subsequent reassembly of a chromosome in a single catastrophic event is truly extraordinary and that, from a purely statistical standpoint, a progressive model is possible. However, a biological explanation for a progressive model was not provided, leaving unexplored as to how DSBs and rearrangements remain locally confined.

Further benefits from the increase in accessibility and use of NGS has facilitated the high-resolution analysis of the break sites that arise during the rearrangements in chromothripsis. This has facilitated more in-depth analyses at the molecular signatures and potential mechanisms involved in the reassembling of the fragments that arise during the chromothriptic shattering event [26,33,49,50,51,52]. Remarkably, these investigations showed no reliance of the reassembly process on large stretches of homology, excluding HR as a primary source of repair. In fact, some fragments were found to display blunt ends, suggesting NHEJ as one repair pathway of choice. In addition, break sites also featured short microhomology tracts of up to 6 bp, with and without short insertions, indicating the involvement of Alt-EJ [26,50,53]. Finally, signs of large insertions (50–500 bp) have also been found and have been reported to be caused by the repair process of microhomology-mediated break-induced replication (MMBIR) [26,33]. In short, multiple DSB repair pathways seem active in the reassembling process after the initiating event in chromothripsis, indicating different possible processes and highlighting the knowledge gap underlying the mechanism(s) of chromothripsis [32,54,55,56,57,58].

## 3. Chromothripsis: Current Models and Their Limitations

### 3.1. Breakage Fusion Bridge Cycle

Though evidence of chromothripsis in human tumors and natural systems is undisputed, many different models have been proposed as to the origin of chromothripsis. Telomeric disfunction was suggested as a model of chromothripsis by Maciejowski et al. by using RPE-1 cells with disrupted telomeric repeat-binding factor 2 (TRF2), leading to telomere fusions and anaphase bridges [59,60]. These anaphase bridges can break when pulled in opposite direction in subsequent mitosis leading to rearrangements and chromosomes that lack telomeres at their broken ends, allowing a cycle of fusion and breakage, termed breakage fusion bridge (BFB) cycle [61]. By sequencing cells that underwent this fusion they found evidence of kataegis and the random reassembly of DNA fragments, a hallmark of chromothripsis [4]. Repetitive BFB events would than give rise to chromothripsis.

### 3.2. Aborted Apoptosis

A different model on the origin of chromothripsis was proposed by Tang et al., where they showed that the reversal of late-stage apoptosis, through the washing away of ethanol as an apoptotic inducer, could cause genomic rearrangements in different cancer cells [36]. Following these findings, Tubio et al. speculated that this process of aborted apoptosis is causally related to chromothripsis in certain cancer cells [35]. After the exposure of stress stimuli or genomic insults some cancer cells start and then abort the apoptotic process, initiating fragmentation of the chromatin through DNases but leaving these cells alive. The most exposed DNA regions would be targeted for cleavage first and could explain the non-random distribution of the breakpoints—an important hallmark of chromothripsis followed by the repair of the cleaved DNA in the surviving cells, which would lead to chromothripsis.

### 3.3. Pulverization of DNA by Exogenous Insults

Finally, different groups have shown that genomic rearrangements can be induced by DNA damage caused by exogenous sources that pulverize part of the DNA, suggesting that this could underly the initiation of chromothripsis. In line with this, Morishita et al. used proton microbeam irradiation to induce DNA DSBs in individual nuclei. They were able to generate monoclonal cell lines form the parental pool that underwent irradiation [37]. WGS of one of these cell lines showed a chromothripsis-like phenotype with complex genomic rearrangements. Other studies showed that exogenous sources of DNA damage, i.e., viral infections, could induce genomic rearrangements [38,62]. Using human papillomavirus to infect human foreskin keratinocytes they showed chromothripsis-like complex rearrangements localized in chromosome eight. However, while virus infections have been associated with viral infections, direct evidence for local pulverization of the chromatin is lacking. Finally, Mardin et al. used topoisomerase II inhibition in hTERT RPE-1 cells to induce DSBs as a way to pulverize the genome and generate chromothriptic cells [63]. Selection and screening of cells with growth advantages and extensive copy number alterations followed by WGS showed massive rearrangements and chromothripsis events. According to the experimental design, these approaches reflect ‘can do’ settings, where multiple break sites lead to chromothripsis, yet this does not exclude alternative more physiological mechanisms.

### 3.4. Micronuclei as Reaction Chamber of Chromothripsis

Despite the fact that DSBs are potent inducers of death signaling [39,40,41,42], most of these models on chromothripsis generally propose that massive chromosomal shattering precedes the rearrangements of the genomic fragments. These scenarios imply that tens to hundreds DSBs simultaneously co-exist within a single cell, without inducing programmed cell death. To prevent DSB-induced stress signals and apoptosis, the level of DSBs was postulated to be at the upper limit of what a cell can tolerate in the presence and absence of the DNA damage response controller TP53 [64]. Considering tens to several hundreds of DSBs, this concept must, in light of the overall cell’s sensitivity to DSBs, be considered unlikely. Though the previously discussed models do not address this issue, one of the more accepted and studied model on the possible origin of chromothripsis proposes that chromosomal shattering takes place in micronuclei [33,34,65], as a solution to this puzzle. Micronuclei form when a chromosome or a chromosome fragment is not incorporated into one of the daughter nuclei during cell division, and are aberrant in DDR signaling, thus facilitating easier breaking of the chromosomes and possibly negating stress signals [66,67,68]. The seminal paper from Peter Ly revealed that this concept of micronuclei-induced chromothripsis, by developing an inducible Y-chromosome centromere-selective inactivation strategy that exploits the CENP-A/histone H3 chimaera. This leads to the abrogation of centromere function and chromosomal segregation of the Y-chromosome. The centromere-inactivated Y-chromosome ends up in a micronucleus, where its fate can be determined in this specialized subcellular compartment [49]. Following this approach, a temporal cascade of events was identified, involving chromosome mis-segregation, fragmentation, and re-ligation spanning three consecutive cell cycles (i–iii). (i) Upon centromere inactivation, a micronucleus harboring the Y-chromosome is formed in the first cell cycle. (ii) Chromosome shattering of the Y-chromosome appeared to be triggered by premature micronuclear condensation prior to or during mitotic entry of the second cycle. (iii) Subsequent canonical NHEJ, but not homology-directed repair, appeared to facilitate the reassembly of chromosomal fragments in the third cell cycle when the content of the micronucleus was reintegrated into the nucleus [49]. While these data indicated that a chromosome can be shattered in a micronucleus, this rarely gave rise to chromothripsis-like structures. In addition, the lack of reliance on microhomology related repair mechanisms suggests the existence of alternative mechanism(s). Other studies in human chromothriptic tumors have indeed implicated DSB repair mechanisms other than NHEJ, which rely on microhomology, such as MMBIR and theta-mediated end joining (TMEJ) [26,69]. In line with this, studies focusing on micronuclei as a reaction chamber for the initiation of complex rearrangements, using various methods to induce the micronuclei, show different results. Zang et al. used nocodazole, a spindle poison, to synchronize cells and induce micronuclei in p53-deficient RPE-1 cells. Live cell microscopy was then applied to identify cells that have reincorporated micronuclei into the main nucleus followed by single cell sequencing. Following this method, large genomic alterations were found displaying evidence of repair signatures indicating alt-EJ and MMBIR as well as NHEJ [33], contrary to Peter Ly et al. Interestingly, a later study conducted by the same group suggested a possible process of the induction of DSBs in these micronuclei [70]. Large amounts of RNA-DNA hybrids were found in micronuclei. Subsequently, RNA:DNA hybrids were found to recruit an enzyme known as adenine deaminases acting on RNA (ADAR), which deaminates adenine to inosine. Next, the base excision repair (BER) pathway was initiated through N-methylpurine DNA glycosylase (MPG), which recognizes and removes these structures, leaving abasic sites. Further processing of these abasic sites by BER resulted in cleavage of the DNA by apurinic/apyrimidinic endonuclease (APE1), creating single-stranded DNA breaks/nick. In large quantities, or when left unresolved during replication, these nicks can lead to DSBs [71,72]. The authors propose this sequence of events as a major cause of the fragmentation in micronuclei leading to chromothripsis. Another study of chromothripsis through micronuclei was conducted by Kneissig et al. using microcell-mediated chromosome transfer to simulate micronuclei formation in RPE-1 cells by causing mitotic slippage in colchicine treated murine A9 donor cells. This spindle poison leads to micronuclei formation, which are then fused with the recipient RPE-1 cells. Interestingly, the analysis of the molecular mechanisms used to reassemble the rearrangements shows some evidence of blunt breakpoint junctions, indicating NHEJ, but the majority of the breakpoints analyzed displayed microhomology based repair signatures [73]. Though these studies show the capability of chromosomal rearrangements to occur through editing in micronuclei, the results of these studies differ substantially in efficiency and in their reliance on the molecular mechanisms used to reassemble the fragments, leaving the option that multiple DSB repair pathways contribute to chromothripsis.

In summary, many different model systems to understand the origin of chromothripsis have been studied, most being initiated through non-physiological conditions. While these ‘Can Do’ conditions provide theoretically possible mechanisms, they leave room for alternative mechanisms.

### 3.5. A Single DSB as Potential Inducer of Chromosomal Rearrangements

**Figure 1:**
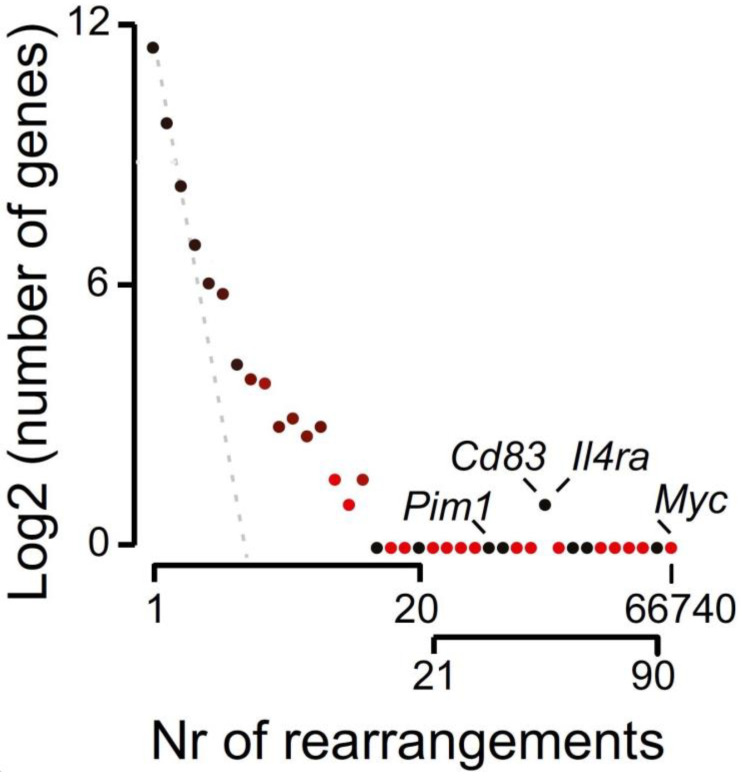
Relation between proximity to a single DSB and rearrangement frequency. Genes in proximity to the primary break site (*Myc* locus) are highly predisposed to becoming involved in a translocation reaction. Translocation events are gradient-colored (red to black), where intrachromosomal (red) are distinguished from interchromosomal (black) translocations. The number of genes with a defined number of rearrangements are grouped and plotted against the number of rearrangements. A small group of genes (e.g., Pim1, IL4ra, and Cd83) are interchromosomal but in fact reside proximal to the DSB. (Adapted from Hogenbirk, M. A. et al. PNAS 2016).

As mentioned, current models on chromothripsis predict that prior to the reshuffling of a chromosome or chromosomal region, tens to hundreds of DSBs have to pre-exist. This requirement extends on a widespread misconception that also governed chromosomal translocations over previous decades: the formation of two or more DSBs precedes chromosomal rearrangements [74,75,76,77,78]. While the co-occurrence of two DSBs with compatible ends, induced by the induction of a rare-cutting mega-nuclease, has been shown to favor chromosomal translocation, the same study indicated that a single DSB induced in the same way was capable of inducing translocations [79]. Furthermore, independent studies based on unbiased genome-wide, molecular and biophysical data have also indicated that chromosomal rearrangements can arise from a single DSB [80,81,82]. In addition, two NGS based technologies, namely, high-throughput genome-wide translocation sequencing (HTGTS) and translocation capture sequencing (TC-Seq), both developed to study chromosome translocations, revealed a preference of mammalian cells to solve a single DSB locally [75,78,83]. HTGTS and TC-SEQ are based on an experimental generation of a single DSB, for example, in a targeted I-SceI site, by a conditionally active or a constitutively active, transduced meganuclease I-SceI, respectively, or alternatively by CRISPR/Cas9. The I-Sce I site is an 18-base pair sequence TAGGGATAACAGGGTAAT, which is not found in the mouse or human genome. Often, when a break is induced, it can be repaired by error prone NHEJ or A-EJ, causing small mutations and thereby destroying the recognition site of the I-Sce I site meganuclease or, in the case CRISR-Cas9-based approaches, the target side of the gRNA. More importantly, in cases where DSBs are not quickly resolved, they can participate in rearrangements with other parts of the genome. With these techniques, these rearrangements can be sequenced and the distance to the original break site quantified. Upon induction of a DSB at a unique I-Sce I site targeted in the *Myc* locus of the mouse genome, a steep downward gradient of rearrangements was observed when plotting the rearrangement frequency versus intragenic, intrachromosomal, and interchromosomal rearrangements (Figure 1). When using a color gradient based on the distance of the rearrangements in relation to the initial DSB, it becomes clear that genomic regions close to the initial break site (red) have a high frequency of being involved in a rearrangement reaction with the initial DSB (intragenic > intrachromosomal). Looking at regions less frequently involved in rearrangement events, these usually involve distal genomic regions, i.e., other chromosomes (intrachromosomal, red > interchromosomal, black). This approach highlighted the preference of the DDR system to resolve a single DSB locally and suggested that accessible chromatin loops in spatial proximity to a DSB are particularly prone to become involved in a rearrangement [83]. Additional evidence of local rearrangements was provided by a FISH-based study confirming that spatial proximity is a key risk determinant for chromosomal translocation [84]. In line with these studies, our own analyses highlighted that in addition to proximity, gene accessibility, rather than gene activity, enhanced the chance of chromosomal rearrangements directly [44]. Taken together, accessible chromatin loops are particularly prone to becoming involved in a rearrangement, which is in line with a recent report highlighting the relevance of the 3D genome structure in the generation of structural variances [85]. This includes DSB-neighboring intrachromosomal genes as well as DSB-neighboring intra- and inter-chromosomal territories [2,35]. Therefore, the frequency by which a genetic element will rearrange is heavily affected by its proximity to the DSB and its accessibility. These notions, as well as independent simulations on genomes affected by chromothripsis by Kinsella et al. [48], predict that chromothripsis could also result from a reaction chain that does not depend on the co-existence of multiple DSBs, but in fact can be initiated and proceeded by a single DSB end.

## 4. The AEG Model of Chromosomal Rearrangements and Chromothripsis

**Figure 2:**
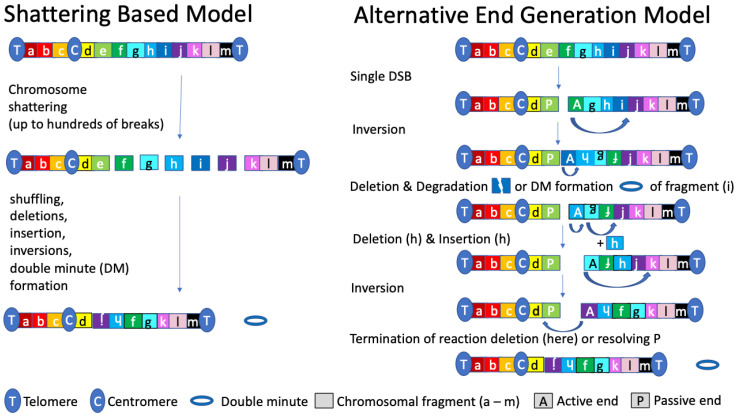
Modeling Chromothripsis: Shattering versus Consecutive Rearrangements. Schematic overview of consecutive AEG reactions leading to multiple different types of rearrangements and the comparison with a shattering-based model. In the shattering model, numerous DSBs are required to enable chromothripsis. In contrast, the AEG model proposes that a single DSB—here, between region e and f—generates a repair-blocking passive end (P) and an intact, i.e., active (A), DNA end. Because the P end blocks DSB repair, the A end is recombined preferentially with proximal accessible elements. This process, which can be associated with deletions, insertions, inversions, and double-minute formation, generates an alternative active end sustaining the domino effect. This catastrophic recombination process stops when the P end is resolved or deleted. While both chromosomal shattering and AEG result in the same chromothripsis product, this viewpoint likes to consider AEG as a model for chromothripsis.

Our integrative genome-wide analyses, aimed to identify direct molecular and biophysical risk determinants of chromosomal rearrangements [44], let us to propose the Alternative End Generations (AEG) model of chromosomal translocations and chromothripsis. This model is based on the fact that uncapped (by specialized proteins) DNA ends are highly reactive, capable of invading and inducing translocations in other parts of the DNA. An example of this is the BFB cycle model, where telomeres break and lack their terminal ends necessary for capping the DNA. These uncapped telomeres fuse with parts of other chromosomes creating the anaphase bridges. A second observation on which this model is based on comes from the fact that large DNA adducts prohibit effective DSB repair. Such adducts need to be removed through various mechanisms before a DNA end can be resolved [86,87,88]. Accordingly, at a DNA DSB, an ‘active’ DNA end (A) that cannot be joined to the ‘passive’, i.e., repair-blocking, DNA end, for instance, because of a DNA lesion (B) has a preference to rearrange with a proximal accessible region in the genome (intragenic > intrachromosomal > interchromosomal) [44]. In this way, an alternative active DNA end, i.e., without a repair-blocking lesion, is generated de novo. If ligated to the pre-existing blocking end, a local rearrangement is generated. If not, alternative ends can be generated consecutively involving predominantly accessible regions. These alternative active ends serve as reaction intermediate favoring the generation of structural variances, such as inversion, deletions, insertions, duplications, and double-minute formation (Figure 2 and graphical abstract). The sequence of these events leading to these different structural variants have been studied and reviewed [89,90]. Consequently, chromosomal rearrangements, including translocations and chromothripsis, can arise as an alternative product of the same event consisting of successive AEG reactions. This model allows for many of the characteristics of chromothripsis and does not have the same difficulties of current models concerning the origin of chromothripsis. For example, given the preference of a single DSB to rearrange proximal to the initial break site [44,83], this model explains why the process underlying chromothripsis often affects only a single chromosome, or a region thereof, and is locally confined. In addition, chromothripsis can be started relatively simply when the initiating DSB is not repaired efficiently, thus omitting the need for a ‘reaction chamber’ like a micronucleus as a specialized compartment to shatter a chromosome, as reported before [33,65]. Furthermore, the AEG model does not rely on the pre-existence of a series of simultaneous DSBs, explaining why chromothripsis escapes severe DNA damage responses and programmed cell death. Finally, tumors that are deficient in one of multiple different DNA repair pathways have been shown to have a high prevalence of chromothripsis [91]. In line with this, the likelihood for a DSB increases, and the formation of a passive, repair-blocking DNA end is more likely to be left unresolved, while the invasion of the active DNA end could potentially be resolved by multiple different repair pathways.

In summary, the AEG model could explain chromothripsis through a multistep reaction process where a single DSB with a repair-blocking end initiates a domino effect of rearrangements that are acquired sequentially in a single catastrophic event, and which is terminated when stable chromatin with only capped, telomeric DNA ends is re-established. The AEG model does not require the pre-existence of many DSBs and shattered chromatin fragments, thereby risking DSB-induced death signaling; it does not depend on chromosomal mis-segregation or rely on the formation of a micronucleus as an isolated shattering compartment. Finally, the AEG model may provide an explanation for the link between DDR defects and chromothripsis, and the localness of the rearrangements in chromothripsis.

## Data Availability

No new data were generated, data shown have been published previously. Please see references.
